# Occupational ocular health problems among marble workers at Shaq El Tho’ban industrial area in Egypt

**DOI:** 10.1007/s11356-021-18410-5

**Published:** 2022-01-23

**Authors:** Enjy A. E. Khorshed, Safaa A. El-Naggar, Samia S. El-Gohary, Ahmed M. B. Awad, Amani S. Ahmed

**Affiliations:** 1 Department of Community, Environmental and Occupational Medicine, Faculty of Medicine, Zagazig University, Zagazig, Egypt; 2 Department of Ophthalmology, Faculty of Medicine, Zagazig University, Zagazig, Egypt

**Keywords:** Occupational, Ocular, Health problems, Marble workers, Shaq El-Tho’ban, Industrial area, Egypt

## Abstract

Eye health of the working population is an essential condition for productivity. Marble industry is processed at large scale at Shaq El Tho’ban area where much dust, crushed pieces of stone, and fluctuating temperatures are endangering employees’ health generally and eye health specifically. The objectives of this study were assessing the prevalence of the most common ocular health problems associated with marble industry and investigating the impact of the working environment and occupational risk factors on the oculo-visual status of marble workers. A cross-sectional study was conducted among 250 workers, working at Shaq El Tho’ban area in Egypt during the period from August 2020 to September 2021, using a semi-structured questionnaire and eye examination comprised of full ocular history, visual acuity testing (unaided/aided), slit lamp examination, ophthalmoscopy, and Schirmer’s type I and tear break up time tests. The current study showed that gritty sensation (65.2%) and eye dryness (51.2%) were the commonest symptoms complained. By examination, conjunctival hyperemia (59.6%) was the most prevalent finding. By performing dry eye tests, dry eye was diagnosed in 60.4% and 51.2% of workers by Schirmer’s test and tear break up time test respectively. The study’s results indicated that age, working category, smoking, and diabetes had significant impact on development of ocular symptoms, while working duration, diabetes, smoking, ocular symptoms, and ocular foreign body had significant impact on development of dry eye disease. Implementation of engineering control measures, proper designing, and supply of eye PPE together with adequate health education to all workers about occupational health risks and preventive measures are recommended.

## Introduction

Marble quarrying and processing industries belong to the oldest industries of the world (Bradley, 2002). Egypt’s marble sector is very competitive and it plays a vital role globally in the socio-economic conditions such as providing employment to thousands of people (Ahmad et al., 2019).

Stone and marble industry had a significant negative impact on public health, including the health of workers directly working in the industry, as well as the health of the population living in the industry’s neighborhoods, in addition to the well-being of the surrounding environment and ecosystems. These impacts result from rock quarrying and crushing activities, large-scale stone cutting and polishing of huge stone in stone-cutting plants, and transportation of rocks, as well as from building and construction’s sites (Salem, 2021). Ocular health problems are among the negative health effects of the marble provided that eyes are the third most common organ affected by injuries apart from the hands and feet (Mir et al., 2014). Occupational eye injuries account for 21% of all workplace injuries of workers in construction, manufacturing and mining who are particularly at risk (Shepherd et al., 2006), and account for 36.7% of injuries according to retrospective epidemiological study in Egypt (Elhesy, 2016), which were higher among male individuals. Furthermore, work-related eye injuries alone cost more than $300 million per year due to loss of production time, medical treatment, and workers’ compensation (OSHA, 2016).

Ocular manifestations range from simple eye strain, minor corneal abrasions up to serious perforating injuries resulting in blindness (Ngo and Leo, 2008). Approximately 90% of these injuries are thought to be preventable (Peate, 2007; Preventblindness, 2016). They are attributable to the misuse or nonuse of protective eyewear by the workers at the time of their work-related ocular injuries (Shashikala et al., 2013).

At the marble processing site, dry, dusty, and heavily polluted environment are identified as the potential occupational hazards; moreover, crushed pieces of stone resulting from marble grinding and polishing are the most common source of injury (Ezisi, 2019). Exposure to airborne marble dust or particles cause irritation of conjunctiva leading to conjunctivitis, itchy, gritty, or foreign body sensations (Gromisch, 2017; Ezisi, 2019; Azuamah et al., 2019). Working environment also may affect, as working in the open areas means being exposed to macroclimatic conditions and solar radiation accentuated by the reflection from the white marble which may affect the workers’ sight, resulting in temporary blindness with major risk of accidents, photoconjunctivitis and photokeratitis, dermatitis, and possible heat stroke during the summer months (Angotzi et al., 2005).

Eye injuries were classified into open-globe and closed-globe injuries based on the Birmingham Eye Trauma Terminology System (Kuhn et al., 2004). Open globe injuries include rupture globe and lacerations. Lacerations subdivide into single laceration (penetrating), two lacerations with entry & exit wounds (perforating) and retained foreign body causing lacerations (intraocular foreign body). While closed globe injuries include superficial foreign body, lamellar lacerations, and contusion (Moore et al, 2017).

The most prevalent ocular problems encountered with marble processing industry and stone workers are intraocular foreign body (Edema et al., 2009), dry eye (Sharma, 2011), pterygium (Koffuor et al., 2012), and corneal abrasions and foreign body (Islam et al., 2000; Qayum et al., 2016).

Wearing adequate protective eyewear is the most important thing for protection of vision at work, which can prevent more than 90% of serious eye injuries (Dang, 2021). There is scarcity of research and data about occupational health problems among marble processing workers in Egypt, so our study aimed at improving occupational health of workers by assessing the prevalence of the most common ocular health problems associated with marble industry and investigating the impact of the working environment and occupational risk factors on the oculo-visual status of marble workers. Our study hypotheses were as follows: (1) null hypothesis, no association between exposure to marble industry and development of ocular symptoms, and (2) alternative hypothesis, there is association between exposure to marble industry and development of ocular symptoms.

## Subjects and methods

A cross-sectional study was carried out among workers at “Shaq El Tho’ban” industrial area at Tura AL-Maadi East Cairo governorate, Egypt, in the period from August 2020 to September 2021. “Shaq El Tho’ban” extends over surface area about 6.5 million m^2^, with workforce of 90,000 of direct workers and indirect workers. It comprises 1858 factories and workshops for manufacturing and exporting marble coming from quarries of Ras Ghareb, Ain El-Sokhna, El Minya, and Jabal Al Galala in Suez, Red Sea, and Aswan (Sis.gov, 2019).

### Study population

#### Determination of sample size and sampling technique

The sample size was calculated through Open-Epi version 3.01, according to the following data: Total population size at “Shaq el Tho’ban” industrial area is 90000 workers (sis.gov, 2019), frequency of eye itchy sensation was about 20% from previous study (Bhatnagar et al., 2014), confidence limit is 5%, design effect = 1, and confidence level = 95%. So the calculated sample size was 245 workers. For better response rate, we added to the sample size 10%, so the final sample size was 269. Participants were selected by simple random sampling technique; they were selected randomly using workers’ list at each workplace with proportional allocation. Pilot study was done using the semi-structured questionnaire on 10% of the sample (25 workers)—who were excluded from the sample—working at one of the factories at “Shaq El-Tho’ban” to test the response to different parts of the questionnaire, to test the feasibility of the study and to recognize the barriers that may arise during data collection. The questionnaire took about 20–30 min, so some workers considered it disrupting their work, so they did not complete it, and others could not identify the problem they suffer from. The results of the pilot study were not included in the results of the study. According to the results of the pilot study, the questionnaire was revised, and some modifications were done, mainly the length of the questions to be concise and removing the questions about the frequency and severity of symptoms as most of participants in pilot study could not determine them.

Inclusion criteria were as follows: adult workers aged 18 years and above, having at least 2 years of work experience, working permanently at the place, and gave their consent to participate in the study.

Exclusion criteria were as follows: Workers who had recent ocular surgery within 1 year, active eye infections, usage of artificial tears within 2 weeks, and wearing contact lenses.

#### Data collection tools

A) Semi-structured questionnaire was used depending on; previous studies (Paz et al., 2013; Occupational Safety and Health Administration (OSHA), 2019) and the Ocular Surface Disease Index (OSDI) questionnaire which is recommended by The International Dry Eye Workshop as a classical and reliable evaluation of dry eye disease (DED) symptoms (Schiffman et al., 2000) which consisted of the following “4 parts”:

Part I: Personal and socio-demographic data: age, level of education, marital status, residence, smoking status.

Part II: Occupational characteristics which included the following: (a) current occupation, (b) years of experience, (c) number of working hours/days, (d) usage of eye personal protective equipment, (e) performance of pre-employment medical examination for workers, (f) performance of periodic medical examination for workers, (g) availability of environmental monitoring, (h) availability of environmental dust control measures, (i) following industrial security. It was translated into Arabic and then validated through a back translation technique and pilot testing.

Part III: General and ocular medical history which included questions about having: (a) hypertension, (b) diabetes, (c) allergy to any substance, (d) previous eye injury during employment, (e) previous eye surgery during employment, (f) previous ocular foreign body during employment.

Part IV: Present history of ocular symptoms: eye burning, eye itching, gritty sensation, eye dryness and blurred vision. It was translated into Arabic and then validated through a back translation technique and pilot testing.

The study`s tools were validated by performing “face validity” which is fulfilled by conducting pilot study and by performing “content validity” which is fulfilled using review of the literature, pilot study, and submitting the questionnaire to a board of occupational medicine experts to judge tool items with relevance and appropriateness.

B) Eye examination

An ophthalmologist examined the workers at the site for eye problems. Full ocular history was taken followed by visual acuity testing (unaided/aided), slit lamp examination, and ophthalmoscopy. Schirmer`s type I and tear break up time tests were done. A portable autorefractometer (Handyref, NIDEK Corp.), portable slit lamp, and Snellen’s chart were used.

##### Schirmer’s test (ST)

It evaluates aqueous tear production. It is helpful in the assessment of patients with signs and/or symptoms of dry eye as it can determine whether surface dryness is due to reduced tear production from the lacrimal glands as opposed to some other causes. The test was performed without anesthetic (Schirmer I) (Eyedocs, 2021).

- Normal value is greater than 10 mm (Shapiro and Merin, 1979).

##### Tear break up time test (TBUT)

It is a clinical test used for assessment of evaporative dry eye disease and measures the stability of tear film.

- Value of < 10 s was considered indicative of tear film instability (Shapiro and Merin, 1979).

## Data management

The collected data were computerized and statistically analyzed using the SPSS program (Statistical Package for Social Science) version 26.0. Frequencies and percentages were used to reflect qualitative data. Mean and standard deviation were used to summarize quantitative results. Median and inter-quartile range was used to describe working experience and Schirmer’s test as data was not normally distributed. Chi-square test was calculated for testing the association between the qualitative data (risk and outcome). Odds ratios (OR) and their 95% confidence intervals (95% CIs) were calculated using univariate logistic regression to measure the association between some sociodemographic, occupational characteristics and medical history with ocular symptoms, and association between some sociodemographic, occupational characteristics and medical, and ocular history with dry eye tests’ results. Paired analysis was done using Wilcoxon signed rank test to compare visual acuity of both eyes among studied participants. Multivariate logistic regression analysis (forward method) was conducted to identify the predictors of each studied outcome. *P* value < 0.05 was considered statistically significant, and *p* value < 0.01 was considered high statistically significant.

## Results

The response rate was 92.9%; total number of 250 workers had participated in the study. All workers were male; most of them (80.4%) were of age (less than 40 years) with a mean of (33.42 ± 8.30 years). Workers (43.6%) had completed either General Secondary Education or Technical Secondary Education Diploma. More than half of them were married (59.2%), living in rural areas (68.4%), and smokers (69.6%).

Regarding occupational characteristics, the working target group was differentiated into categories: 17.2% of workers were of administrative category comprising (engineers, production managers, and production supervisors), 32.4% were of marble processing category comprising (crane operators and assistants, polishing workers and assistants, and saw machine workers and assistants), 26.4% of the supportive category comprising (machinery technicians, electrical technicians, drivers, carpenters, and packing and loading workers), and 24% were of the installation and ornamental category workers. Most of the workers (78.4%) had less than 20 years of experience with range (2–37 years). More than half of the workers (75.2%) worked for 8 h/day, and none of them was using eye PPE. None of participants had neither pre-employment nor periodic medical examination. All the workers reported unavailability of environmental monitoring and local exhaust ventilation system (dust control measures), and all were not following industrial security. Regarding general medical history, hypertension was reported among (20.8%) of workers, while diabetes was identified among (18.0%). About ocular history, encountering ocular foreign body was reported among 47.6% of participants, 10.8% had eye injuries, and only 8.0% had undergone previous eye surgery (Table 1). None of the participants was using eye PPE mostly due to unavailability (62%) and the rest due to unawareness of its benefit, poor vision with the device, and feeling discomfort (18%, 11.2%, and 8.8% respectively).

**Table 1:** Socio-demographic, occupational characteristics & medical history of the studied participants

Items	*N* = 250	%
Age (X` ± SD) (Range) 20- 30- ≥ 40	(33.42 ± 8.30) (20–54)	
	91 110 49	36.4 44.0 19.6
Education		
Illiterate and primary Preparatory Secondary/diploma University or higher	23 82 109 36	9.2 32.8 43.6 14.4
Marital status		
Married Unmarried	148 102	59.2 40.8
Residence		
Rural Urban	171 79	68.4 31.6
Smoking status		
Smoker Non-smoker	174 76	69.6 30.4
Working category		
Administrative workers Marble production workers Supportive workers Installation and ornamental workers	43 81 66 60	17.2 32.4 26.4 24.0
Working experience (years)		
Median (Range)	14 (2–37)	
**˂ **10 10- ** ≥ **20	84 112 54	33.6 44.8 21.6
No. of working hours/day		
8 h 10 h	188 62	75.2 24.8
General medical history		
Hypertension Diabetes Allergy to any substance	52 45 7	20.8 18.0 2.8
Ocular medical history		
Eye surgery Eye injury Ocular foreign body	20 27 119	8.0 10.8 47.6

**Fig. 1:**
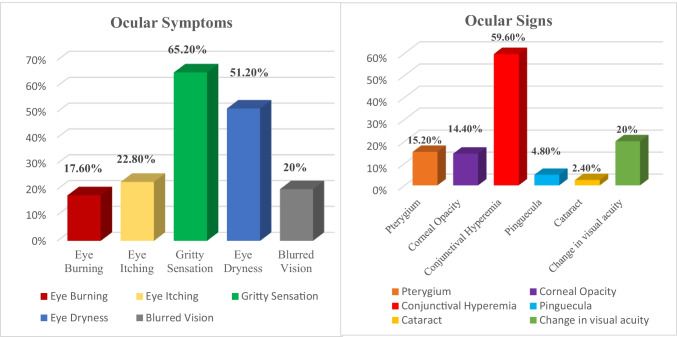
Ocular symptoms and signs among the studied participants

Important ocular symptoms were identified among 134 (53.6%) of the participants, while 116 (46.4%) did not show any ocular symptoms. Gritty sensation (65.2%) and eye dryness (51.2%) were the commonest symptoms followed by eye itching (22.8%), blurred vision (20.0%), and eye burning (17.6%). By examination, the following signs were detected, conjunctival hyperaemia (59.6%) was the most prevalent finding, followed by change in visual acuity (20.0%), pterygium (15.2%), corneal opacity (14.4%), pinguecula (4.8%), and cataract (2.4%)was the least prevalent sign detected (Fig. 1).

**Fig. 2:**
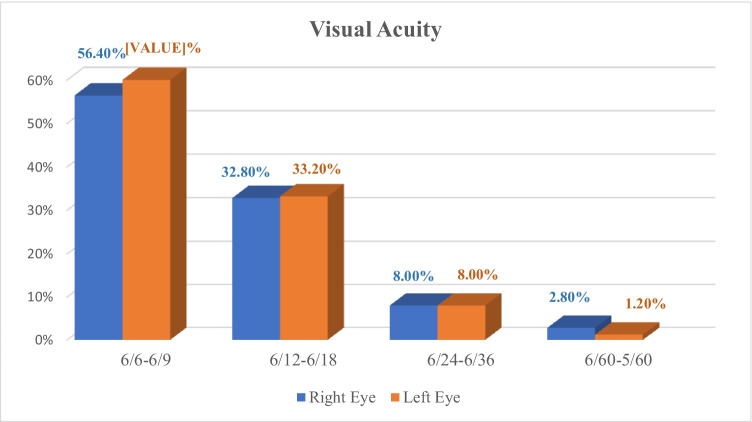
Visual acuity of right and left eyes of the studied participants

The visual acuity (VA) of the participants was taken monocularly. VA of left eyes was significantly better than VA of right eyes (*p* = 0.001) which was analyzed by Wilcoxon signed rank test (Fig. 2).

Factors that significantly increased risk of occurrence of ocular symptoms among the studied participants were workers’ age of 30 to less than 40 years and ≥ 40 years, smoking status, secondary/diploma education, working duration for 20 years or more, working in marble processing category, and finally having diabetes (Table 2).

**Table 2:** Relation between some sociodemographic, occupational characteristics and medical history and ocular symptoms of the studied participants

	Ocular symptoms				COR (CI 95%)
	Present *N* = 134		Absent *N* = 116		
	No	%	No	%	
Age of workers					
20- (*n* = 91) 30- (*n* = 110) ≥ 40 (*n* = 49)	34 67 33	37.4 60.9 67.3	57 43 16	62.6 39.1 32.7	1.0 2.6 (1.47–4.62)** 3.45 (1.66–7.19)**
Education:					
Illiterate and primary (*n* = 23) Preparatory (*n* = 82) Secondary/diploma (*n* = 109) University or higher (*n* = 36)	8 45 64 17	34.8 54.9 58.7 47.2	15 37 45 19	65.2 45.1 41.3 52.8	1.0 2.28 (0.87–5.9) 2.66 (1.04–6.81)* 1.67 (0.57–4.9)
Residence					
Rural (*n* = 171) Urban **(***n* = 79)	89 45	52.0 57.0	82 34	48.0 43.0	1.21 (0.71–2.08)
Marital status					
Married (*n* = 148) Unmarried (*n* = 102)	85 49	57.4 48.0	63 53	42.6 52.0	0.685 (0.41–1.13)
Smoking status					
Smoker (*n* = 174) Non-smoker (*n* = 76)	105 29	60.3 38.2	69 47	39.7 61.8	2.46 (1.41–4.29)**
Working category					
Administrative workers (*n* = 43) Marble processing workers (*n* = 81) Supportive workers (*n* = 66) Installation and ornamental workers (*n* = 60)	18 54 26 36	41.9 66.7 39.4 60.0	25 27 40 24	58.1 33.3 60.6 40.0	1.0 2.77 (1.29–5.95)** 0.90 (0.41–1.97) 2.08 (0.93–4.6)
Working duration (years)					
˂ 10 (*n* = 84) 10- (*n* = 112) ≥ 20 (*n* = 54)	37 59 38	44.0 52.7 70.4	47 53 16	56.0 47.3 29.6	1.0 1.41 (0.80–2.49) 3.01 (1.46–6.23)**
General medical history					
Hypertension					
Yes (*n* = 52) No (*n* = 198)	31 103	59.6 52.0	21 95	40.4 48.0	1.36 (0.73–2.53)
Diabetes					
Yes (*n* = 45) No (*n* = 205)	38 96	84.4 46.8	7 109	15.6 53.2	6.16 (2.63–14.4)**
Allergy to any substance	6	85.7	1	14.3	
Yes (*n* = 7) No (*n* = 243)	128	52.7	115	47.3	5.39 (0.63–45.44)

*Statistically significant *p* value < 0.05.

**High statistically significant *p* value < 0.01.

In table 3, factors detected by Schirmer`s test that significantly increased risk of developing dry eye were workers` age of ≥ 40 years, preparatory & secondary/diploma education, urban residence, smoking status, working in marble processing category, working duration of 10 to less than 20 years and for ≥ 20 years, having ocular symptoms, hypertension and diabetes and past history of ocular foreign body and eye injury. While factors detected by tear break up time test that significantly increased risk of developing dry eye were workers` age of 30 to less than 40 and ≥ 40 years, all educational levels above primary education, urban residence, marital status, smoking status, working duration of 10 to less than 20 years and for ≥ 20 years, having ocular symptoms, hypertension and diabetes and past history of ocular foreign body and eye injury.

**Table 3:** Relation between some sociodemographic, occupational characteristics, medical, and ocular history and dry eye tests’ results*

	Schirmer’s test results				OR (CI 95%)	Break Up test results				COR (CI 95%)
	Normal *N* = 99		Dry Eye *N* = 151			Normal *N* = 122		Dry eye *N* = 128		
	No	%	No	%		No	%	No	%	
Age of workers										
20- (*n* = 91) 30- (*n* = 110) ≥ 40 (*n* = 49)	43 42 14	47.3 38.2 28.6	48 68 35	52.7 61.8 71.4	1.0 1.45 (0.82–2.54) 2.23 (1.06–4.71)*	57 48 17	62.6 43.6 34.7	34 62 32	37.4 56.4 65.3	1.0 2.16 (1.22–3.8)** 3.15 (1.5–6.5)**
Education										
Illiterate and primary (*n* = 23) Preparatory (*n* = 82) Secondary/diploma(*n* = 109) University or higher(*n* = 36)	15 27 40 17	65.2 32.9 36.7 47.2	8 55 69 19	34.8 67.1 63.3 52.8	1.0 3.81 (1.44–10.11)** 3.23 (1.26–8.29)* 2.09 (0.71–6.16)	19 36 52 15	82.6 43.9 47.7 41.7	4 46 57 21	17.4 56.1 52.3 58.3	1.0 6.06 (1.8–19.4)** 5.20 (1.66–16.3)** 6.65 (1.87–23.5)**
Residence										
Rural (*n* = 171) Urban **(***n* = 79)	82 17	48.0 21.5	89 62	52.0 78.5	3.36 (1.81–6.21)**	97 25	56.7 31.6	74 54	43.3 68.4	2.83 (1.61–4.96)**
Marital status										
Unmarried (*n* = 102) Married (*n* = 148)	47 52	46.1 35.1	55 96	53.9 64.9	1.57 (0.94–2.64)	59 63	57.8 42.6	43 85	42.2 57.4	1.85 (1.11–3.08)
Smoking status										
Non-smoker (*n* = 76) Smoker (*n* = 174)	53 46	69.7 26.4	23 128	30.3 73.6	6.4 (3.54–11.61)**	51 71	67.1 40.8	25 103	32.9 59.2	2.95 (1.68–5.2)**
Working category										
Administrative (*n* = 43) Marble processing (*n* = 81) Supportive (*n* = 66) Installation and ornamental (*n* = 60)	19 21 39 20	44.2 25.9 59.1 33.3	24 60 27 40	55.8 74.1 40.9 66.7	1.0 2.26 (1.03–4.93)* 0.54 (0.25–1.19) 1.58 (0.70–3.54)	21 29 43 29	48.8 35.8 65.2 48.3	22 52 23 31	51.2 64.2 34.8 51.7	1.0 0.53 (0.25–1.12) 0.51 (0.23–1.11) 1.02 (0.46–2.23)
Working duration (years)										
˂ 10 (*n* = 84) 10- (*n* = 112) ≥ 20 (*n* = 54)	49 39 11	58.3 34.8 20.4	35 73 43	41.7 65.2 79.6	1.0 2.62 (1.46–4.69)** 5.47(2.47–12.07)**	57 51 14	67.9 45.5 25.9	27 61 40	32.1 54.5 74.1	1.0 2.52 (1.39–4.55)** 6.03 (2.8–12.9)**
Ocular symptoms Absent (*n* = 116) Present (*n* = 134)	70 29	60.3 21.6	46 105	39.7 78.4	5.51(3.16–25.1)**	75 47	64.7 35.1	41 87	35.3 64.9	3.38 (2.01–5.69)**
General medical history										
Hypertension										
Yes (*n* = 52) No (*n* = 198)	7 92	13.5 46.5	45 106	86.5 53.5	5.58 (2.39–12.97)**	17 105	32.7 53.0	35 93	67.3 47.0	2.32 (1.22–4.42)**
Diabetes										
Yes (*n* = 45) No (*n* = 205)	5 94	11.1 45.9	40 111	88.9 54.1	6.77 (2.57–17.8)**	14 108	31.1 52.7	31 97	68.9 47.3	2.46 (1.23–4.9)**
Allergy to any substance										
Yes (*n* = 7) No (*n* = 243)	1 98	14.3 40.3	6 145	85.7 59.7	4.05 (0.48–34.2)	1 121	14.3 49.8	6 122	85.7 50.2	5.95 (0.7–50.1)
Ocular medical history										
Ocular foreign body										
Yes (*n* = 119) No (*n* = 131)	26 73	21.8 55.7	93 58	78.2 44.3	4.50 (2.58–7.84)**	40 82	33.6 62.6	79 49	66.4 37.4	3.30 (1.9–5.5)**
Eye injury										
Yes (*n* = 27) No (*n* = 223)	5 94	18.5 42.2	22 129	81.5 57.8	3.20 (1.17–8.77)*	7 115	25.9 51.6	20 108	74.1 48.4	3.04 (1.23–7.48)*
Eye surgery										
Yes (*n* = 20) No (*n* = 230)	8 91	40.0 39.6	12 139	60.0 60.4	0.98 (0.38–2.49)	8 114	40.0 49.6	12 116	60.0 50.4	1.47 (0.58–3.74)

*Statistically significant (*p* value ˂ 0.05).

**High statistically significant (*p* value < 0.01).

Table 4 shows that age, smoking status, working category, and diabetes mellitus were the significant predictors of occurrence of ocular symptoms. Working duration, presence of ocular symptoms, smoking status, DM, and ocular FB were the significant predictors of developing dry eye detected by Schirmer’s test, while working duration, presence of ocular symptoms, and ocular FB were the significant predictors of developing dry eye detected by break up time test.

**Table 4:** Binary logistic regression analysis for the significant predictors of occurrence of ocular symptoms, dry eye by Schirmer’s test, and break up time test

	B	S.E	Wald	AOR (CI 95%)
I: **Predictors of occurrence of ocular symptoms**				
Age				
20- 30- ≥ 40	0.772 2.131	0.364 0.606	12.50 4.49 12.35	1.0 2.16 (1.06–4.41)* 8.42 (2.56–27.6)**
Smoking status	1.095	0.325	11.36	2.98 (1.58 – 5.64)**
Working category				
Administrative Marble processing Supportive Installation and ornamental	2.484 1.706 1.391	0.855 0.829 0.846	12.78 8.43 4.23 2.70	1.0 11.99 (2.24–64.12)** 5.50 (1.08–27.9)* 4.02 (0.76–21.11)
DM	1.309	0.489	7.173	3.70 (1.42–9.65)**
**II: Predictors of dry eye by Schirmer’s test**				
Working duration				
˂ 10 10- ≥ 20	0.836 1.420	0.385 0.510	8.86 4.72 7.75	1.0 2.30 (1.086–4.90)* 4.13 (1.52–11.23)**
Presence of ocular symptoms	1.551	0.350	19.75	4.73 (2.38–9.39)**
Smoking status	1.622	0.373	18.90	5.06 (2.43–10.5)**
DM	1.730	0.652	7.06	5.66 (1.57–20.3)**
Ocular FB	1.501	0.363	17.15	4.50 (2.20–9.17)**
**III: Predictors of dry eye by break up time test**				
Working duration				
˂ 10 10- ≥ 20	0.725 1.813	0.329 0.423	18.492 4.839 18.409	1.0 2.06 (1.08–3.93)* 6.12 (2.67–14.02)**
Presence of ocular symptoms	1.092	0.291	14.026	2.97 (1.68–5.27)**
Ocular FB	1.350	0.299	20.367	3.85 (2.14–6.93)**

*Statistically significant *p* value < 0.05.

**High statistically significant *p* value < 0.01.

## Discussion

“Shaq El Tho’ban” industrial area has been known for marble industry on international wide scale, but this was found to be associated with ocular health problems among marble workers. In our study, all the workers were males; this could be attributable to the physical nature of work of marble industry in which females in Egypt are not involved in this nature of work. That was in accordance with Okoye and Umeh (2000) as all workers were males in their study. The age distribution indicates that most workers were young or middle aged, which was similar to another study carried out in Ghana (Koffuor et al., 2012).

Most of the workers (78.4%) worked for less than 20 years that agreed with Tetteh et al. (2020) in which (37.4%) of workers had working experience of more than 10 years but contrasts with report by Ezisi et al. (2017) where workers had a working experience of only 5 years or less. Workers who had spent multiple years in the industry are less likely to encounter work-related injuries because they are experienced, more mature, and more aware about workplace hazards. Unfortunately, none of the workers of our study was using eye PPE mostly due to unavailability (62%) and unawareness of its benefit (18%). As reported by Ezisi et al. (2017), only (1.3%) of the participants used eye goggles irregularly and the others neglected using it due to unavailability (76.1%) and high cost (10.6%). On the contrary (Koffuor et al., 2012) noted in their study, that (45.7%) of workers used some form of protective eyewear (either sunglasses or safety goggles). This result can be attributed to that (42%) of workers did not finish secondary education and may have inadequate knowledge about health hazards they are exposed to at marble industry without wearing personal protective equipment (PPE) and the employers did not provide them with the PPE they need at some workplaces. That was in accordance with similar studies which have reported that industrial workers in developing countries lack the knowledge and awareness of the health effects of exposure to work site hazards (El-Sersy, 2014; Ramesh and Joseph 2015).

None of the participants had pre-employment ocular examination that was nearly similar to a study of stone quarry workers’ awareness of work-related ocular health hazards in Nigeria (Ezisi et al., 2017); as among 500 workers, only 7 of them had pre-employment examination (1.4%); also, Okoye and Umeh (2000) reported that the vast majority in their study did not undergo pre-employment test, which indicates much negligence of employers concerning implementation of occupational safety and health practices specially in developing countries. Unavailability of environmental monitoring and dust control measures, besides all workers not following industrial security, revealed absence of governmental supervision and employers` dereliction.

Regarding general and ocular medical history, work-related eye-injury was notified only among 10.8% of workers of our study which were of age group 30 to less than 40 years. On contrast, there were 32.8% of workers having work-related eye injuries as notified by Ezisi et al. (2017). Also, on a study of risk factors for work-related eye injuries among stone quarry workers (Ezisi, 2019), 64.1% of workers had work-related eye injuries, more injuries occurred in the younger age group, and most of them were working for 5 years or less. There was conflict between the previous studies and Kanoff et al. (2010) reporting higher injury occurrence among older age group mainly from inattention and easy fatigability. This wide variation between our study and the other studies emphasizes that longer work experience reduces incidents of work-related eye injury and ensures proficiency. History of ocular foreign body was identified among 47.6% of workers that was in accordance with Ezisi (2019) where foreign body injuries affected 58.2% of stone workers and were the most common type of injuries. This was consistent with Edema et al. (2009) who also identified foreign body as the most common type of eye injury in industrial workers as general.

Important ocular symptoms were identified among the participants: eye burning, eye itching, gritty sensation, eye dryness, and blurred vision. This appears reasonable as most of the workers were in direct or indirect contact with particulate matter and dusty environment that annoy senses causing irritation even other than ocular. This can explain that most workers were noticed to have a behavior of massive irrigation of affected eyes with clean water following excessive contact. It is well known that gritty sensation is one of the symptoms of eye dryness, but usually people describe their complain in this term (dry eye) which they consider it different from (gritty sensation) that they use to describe foreign body sensation.

Similar ocular symptoms were identified among stone workers in Nigeria (Azuamah et al., 2019) as itching, irritation, sandy sensation, and blurry vision. Also, in Ghana (Koffuor et al., 2012), sand and stone workers complained of burning sensation, itchy sensation, gritty sensation, blurred vision, and photophobia.

By examination, conjunctival hyperemia (59.6%) was the most prevalent finding as marble dust is recognized a potential occupational hazard at marble processing site causing ocular irritation predisposing workers to conjunctivitis. This can be supported by Rohatgi (2013) who reported conjunctival hyperemia as a consequence of long-term exposure to particulates among stone workers.

Other signs include change in visual acuity, pterygium, corneal opacity, pinguecula, and cataract. As supported by Good Vision for Life (2020), pterygium and pinguecula could result from exposure to excessive UV radiation from sunlight, dust, and fumes in the environment which resemble the marble industry environment. This corresponds with findings of Al-Bdour and Al-Latayfeh (2014) that showed a strong positive association between outdoor work and the development of pterygium and pinguecula. Similarly, another study among stone workers Azuamah et al. (2019) identified pterygium, pinguecula, cataract, and conjunctival hyperemia. Also, Isawumi et al. (2011) and Erdoğan et al. (2011) recognized pterygium and cataract as two major ocular problems among stone workers. Aliyu and Shehu (2006) reported corneal opacity among stone quarry workers who did not wear their safety goggles. Moreover, Mantyjarvl (2000) reported that cataract was a major problem found among industrial workers and it reduced their visual acuity and contrast sensitivity.

Although examination showed that many workers had normal visual acuity indicating that the working environment had minimal effect on the visual status of the workers, there were workers with best-corrected visual acuity but did not wear glasses for fear of getting broken or dusting of glasses by marble dust and cannot afford doing refractive surgery or contraindicated due to eye dryness. Best-corrected visual acuity means the measurement of the best vision correction as measured on the standard Snellen eye chart that can be achieved using glasses or contact lenses.

According to the recommendation of the 2007 Report of International Dry Eye Workshop (Alshamrani et al., 2017), to combine subjective symptoms with objective clinical tests to confirm dry eye diagnosis, we performed tests of tear function to confirm the symptoms complained by workers which suggest dry eye disease (DED). Prevalence of DED was found to be 60.4% diagnosed by Schirmer’s test (ST) indicating lack of aqueous tear secretion by the lacrimal glands while by break up time test (TBUT) was 51.2% indicating tear film instability. However, Alshamrani et al. (2017) reported prevalence of (32.1%) in Saudi Arabia, which was lower than the prevalence reported by our study. This variation results from many factors; the most important is different target groups as our participants were workers, while in the other study, they were public population not exposed to the same climatic conditions and occupational risk factors our participants were exposed to that certainly had affected their ocular status.

There were multiple risk factors that significantly increased risk of developing ocular symptoms and dry eye; age of workers was a significant risk factor as with increasing age; the risk of developing ocular symptoms and dry eye detected by both ST and TBUT increased. That agrees with de Paiva (2017) which declared that aging is a significant risk factor for dry eye, and according to Schaumberg et al. (2009), tear dysfunction has been found to increase with age from the 4th to 8th decade of life. Also, proved by (Sledge et al., 2017) that tear film stability decreases with advancing age which manifests as decreased value of tear break up time. Educational levels higher than primary education were significant risk factor for developing ocular symptoms and dry eye detected by both low ST and TBUT values; this can be explained that educated workers feel self-confident or arrogant that make them not interested or careless about commitment to safety precautions, using eye PPE and guarding against workplace hazards.

Urban residence was a significant risk factor of developing dry eye more than rural residence, and their prevalence were 78.5% and 68.4% detected by low ST and TBUT values respectively, while residence had no effect on ocular symptoms development. Similarly, Titiyal et al. (2018) and Donthineni et al. (2019) found that 65.02% and 59% of dry eye patients respectively were from urban localities. This can be justified by multiple sources of air pollution in densely populated cities which are considered risk factors of DED as automobiles and industrial facilities Saxena et al. (2003). Moreover, Aziz and Tawfik (2020) noticed that urban areas where they conducted their study were contributing factors to the more air pollution exposure than the other studied populations. Additionally, Gupta et al. (2002) demonstrated the effect of air pollution on DED in urban areas of Delhi, where tear film instability occurred after long-term exposure to atmospheric pollutants.

In our study, smoking was a highly significant risk factor of developing ocular symptoms and dry eye detected by low ST and TBUT values; also in Titiyal et al. (2018), cigarette smoking was identified as a significant risk factor (OR = 1.5) for severe dry eye disease. Besides (Aziz and Tawfik (2020) recorded statistically significant association between DED and smoking. As proved by (Altinors et al., 2006) who reported that smoking damages the lipid layer of the precorneal tear film by process of lipid peroxidation causing tear film instability, leading to symptoms of DED.

Workers in marble processing category were 3 times (OR = 2.77) at risk of developing ocular symptoms and 2 times (OR = 2.26) at risk of developing dry eye detected by low ST values more than other working categories. This can be explained by the working conditions of  marble processing workers compared to other working categories as they are directly and continuously exposed to the process and multiple risk factors as dust, crushed pieces of stone and heavily polluted environment (during loading/unloading of huge blocks by crane operators, marble cutting using diamond wires or a gang saw under water stream or electric saw in workshops or during polishing either automatic or manual) which led to development of those symptoms and affection of aqueous tear production by lacrimal glands. While working category had no significant association with low TBUT values which indicates that working category is not significantly associated with tear film instability.

Working duration for 20 years or more was a significant risk factor of developing ocular symptoms and dry eye detected by both low ST and TBUT values; this is reasonable due to prolonged continuous exposure to multiple hazards at workplace, so with increasing duration of exposure, risk of developing ocular symptoms and dry eye increased.

Hypertensive workers were at risk of dry eye detected by both low ST and TBUT values; this can be explained by the finding of Akcay et al. (2015) that the use of anti-hypertensive drugs containing ACE (angiotensin converting enzyme) inhibitors used in treatment of hypertension can cause DED. Also, Al Houssein et al. (2017) documented in their study that prevalence of dry eye syndrome in hypertensive patients was 48% which support our findings. Diabetic workers also were at risk of developing ocular symptoms and dry eye detected by both low ST and TBUT values; this can be supported by Jeganathan et al. (2008) who declared a range of ocular diseases associated with diabetes rather than diabetic retinopathy with subsequent ocular symptoms (blurry vision, conjunctival hyperemia, eye itching). It also agrees with Ozdemir et al. (2003) who found that TBUT and ST values were significantly lower in diabetic patients and Kamel et al. (2017) who concluded that diabetic patients are more prone to suffering from dry eye than normal people as diabetes and dry eyes appeared to be linked.

In addition, diabetes has also been identified as one of the most important systemic risk factors for DED (Zhang et al., 2016).

Ocular foreign body was identified to be a highly significant risk factor of dry eye detected by both low ST and TBUT values; this can be justified by corneal abrasions caused by corneal foreign bodies that lead to alterations in tear production. This agrees with Hegarty et al. (2018) who found that abrasion would affect tear production at time following injury in the form of extensive observed blinking that may have produced reflex tears that would have masked a decrease in normal tear production caused by corneal nerve damage.

Eye injury was found to be significant risk factor of dry eye detected by both low ST and TBUT values; this concurs with Ang et al. (2001) who reported that corneal abrasions can trigger dry eye symptoms, including defects in tear production.

By multivariate logistic regression analysis, workers’ age of 30 to less than 40 years and ≥ 40 years, marble processing and supportive working categories, smoking, and diabetes were the most important significant predictors for development of ocular symptoms among marble workers. Some of supportive category workers (machinery technicians, electrical technicians) were working at marble processing site together with marble processing workers and exposed to the same hazards continuously, so they were at less risk of developing ocular symptoms (adjusted OR = 5.5) caused by marble dust and airborne particulates, while marble processing workers were at greater risk (adjusted OR = 11.99). Regarding dry eye, regression showed that working duration of 10 to less than 20 years and ≥ 20 years, diabetes mellitus, presence of ocular symptoms, and history of ocular FB were the most significant predictors for dry eye detected by Schirmer’s test, while the most significant predictors for dry eye detected by break up time test were the same as ST except for smoking and diabetes. Furthermore, data suggests that past history of ocular FB (which is common among workers at marble industry) was a significant predictor of DED among marble workers.

## Conclusion

Although the work environment of marble industry almost had minor impact on visual acuity, it has significant impact on ocular function which later could affect the vision. According to the current study, workers suffer from variety of ocular problems mostly eye dryness and conjunctival hyperemia, which is a common sign of acute anterior inflammation, that comply with lack of environmental dust control measures at workplace besides non-usage of eye PPE which could have prevented or minimized these ocular problems.

## Recommendations

Implementation of engineering control methods such as enclosure of dust-producing processes under negative air pressure (minor vacuum compared to the air pressure outside the enclosure) and installation of local exhaust air ventilation system which removes air containing dust through a collection system before releasing it into the atmosphere. For the safety of workers, cutting and polishing machinery must be improved. Employers should supply eye PPE that is properly designed to suit the climatic working conditions and reduce discomfort of the workers. Before being hired, workers should have a pre-employment medical examination and should undergo periodic screening. Regular examination of diabetic workers is indicated for detecting ocular surface disorders.

Workers should have information about health risks, appropriate working conditions, personal hygiene, and preventive measures. Prospective longitudinal research in environmental and occupational medicine is required to improve the perception and diagnostic approach to ocular damage caused by environmental and occupational factors which may help in development of preventive measures for preventing occupational diseases and in using occupational surveillance to counteract advancing age-related ocular impairment.

## Study limitations

Some of the crucial variables, such as the history of an ocular foreign body or an eye injury, may be influenced by information bias or recall bias. Non-performance of pre-employment eye tests for all workers made it difficult or even impossible to determine their pre-employment ocular status which would have served as a baseline for comparison. If such records had been available, reports of any workplace industrial injuries would have been confirmed. There was no comparison group to use.

## Data Availability

The datasets used and analyzed during the current study are available from the corresponding author on reasonable request.

## References

[CR1] Ahmad Z, Khan SM, Ali MI, Fatima N, Ali S (2019) Pollution indicium and marble waste polluted ecosystem; role of selected indicator plants in phytoremediation and determination of pollution zones. J Clean Prod 236–117709

[CR2] Akcay EK, Akcay M, Can GD, Aslan N, Uysal BS, Ceran BB, Koseahya P, Cagil N (2015). The effect of antihypertensive therapy on dry eye disease. Cutan Ocul Toxicol.

[CR3] Al Houssein AO, Al Houssein RO, Al Hawass AA (2017) Magnitude of diabetes and hypertension among patients with dry eye syndrome at a tertiary hospital of Riyadh, Saudi Arabia- A case series. Saudi J Ophthalmol:31(2):91- 94. 10.1016/j.sjopt.2017.02.001 10.1016/j.sjopt.2017.02.001 PMC5436380 28559720

[CR4] Al-Bdour MD, Al-Latayfeh MM (2014). Risk factors for pterygium in an adult Jordanian population. Acta Ophthalmol Scand.

[CR5] Aliyu AA, Shehu AU (2006). Occupational hazards and safety measures among stone quarry workers in northern Nigeria. Niger Med Pract.

[CR6] Alshamrani AA, Almousa AS, Almulhim AA, Alafaleq AA, Alosaimi MB, Alqahtani AM, Almulhem AM, Alshamrani MA, Alhallafi AH, Alqahtani IZ, Alshehri AA (2017). Prevalence and Risk Factors of Dry Eye Symptoms in a Saudi Arabian Population. Middle East Afr J Ophthalmol.

[CR7] Altinors DD, Akça S, Akova YA, Bilezikçi B, Goto E, Dogru M (2006). Smoking associated with damage to the lipid layer of the ocular surface. Am J Ophthalmol.

[CR8] Ang RT, Dartt DA, Tsubota K (2001). Dry eye after refractive surgery. Curr Opin Ophthalmol.

[CR9] Angotzi G, Bramanti L, Tavarini D, Gragnani M, Cassiodoro L, Moriconi L, Saccardi P, Pinto I, Stacchini N, Bovenzi M (2005) World at work: *Marble quarrying in Tuscany*. Available from: https://www.researchgate.net/publication/7839507_World_at_work_Marble_quarrying_in_Tuscany [accessed Dec 22 2020] 10.1136/oem.2004.018721 PMC1741029 15901891

[CR10] Aziz BF, Tawfik CA (2020). Prevalence of dry eye disease among healthy Egyptian population. J Egypt Ophthalmol Soc.

[CR11] Azuamah YC, Nwazunku A, Amadi AN, Esenwah EC, Ikoro NC, Megwas AU (2019) Major ocular problems found among quarry workers and residents of quarrying communities in Abakaliki, Southeastern Nigeria Available from: https://www.researchgate.net/publication/334119478_Major_ocular_problems_found_among_quarry_workers_and_residents_of_quarrying_communities_in_Abakaliki_Southeastern_Nigeria [accessed Sep 30 2020].

[CR12] Bhatnagar KR, Sapovadia A, Gupta D, Kumar P, Jasani H (2014). Dry eye syndrome: a rising occupational hazard in tropical countries. Med J DY Patil Univ.

[CR13] Bradley F (2002) The Stone Industry Worldwide: Current State and Trends, Marble-Stat 2002. Associazione Italiana, Panorama

[CR14] Dang Sh (2021). Prevent Workplace Eye Injuries During COVID-19. American Academy of Ophthalmology (aao.org). Available from: https://www.aao.org/eye-health/tips-prevention/injuries-work

[CR15] de Paiva CS (2017). Effects of Aging in Dry Eye. Int Ophthalmol Clin.

[CR16] Donthineni PR, Kammari P, Shanbhag SS, Singh V, Das AV, Basu S (2019). Incidence, demographics, types and risk factors of dry eye disease in India: Electronic medical records driven big data analytics report I. Ocul Surf.

[CR17] Edema OT, Omoti AE, Akinsola FB, Aigbotsua P (2009). Ocular injuries in industrial technical workers in Delta State, Nigeria. J Hainan Med College.

[CR18] Elhesy AE (2016). Retrospective study of ocular trauma in Mansoura Ophthalmic Center. J Egypt Ophthalmol Soc.

[CR19] El-Sersy TH (2014). Role of pterygium in ocular dryness. J Egy Ophthalmol Soc.

[CR20] Erdoğan H, Ozdimir L, Astram S, Ozec A, Cetinya S, Sümer H (2011). Prevalence of refraction errors and color blindness in heavy vehicle drivers. Int J Ophthalmol.

[CR21] Eyedocs (2021). Schirmer's test. Available at: https://www.eyedocs.co.uk/ophthalmology-articles/cornea/505-schirmers-test (Accessed 20 May 2021)

[CR22] Ezisi CN (2019) Risk factors for work-related eye injuries among stone quarry workers: a field report. Niger J Ophthalmol; 27:33–40. Available from: http://www.nigerianjournalofophthalmology.com/text.asp?2019/27/1/33/262064

[CR23] Ezisi CN, Eze BI, Okoye O, Arinze O (2017). Correlates of stone quarry workers' awareness of work-related ocular health hazards and utilization of protective eye devices: Findings in southeastern Nigeria. Indian J Occup Environ Med.

[CR24] Good Vision for Life (2020). Pterygium symptoms, causes, risks & treatment. Available from: https://goodvisionforlife.com.au/vision-problems/pterygium/ (accessed Jun 2021).

[CR25] Gromisch M (2017) Health Risks of Marble Dust . Available from: https://oureverydaylife.com/226992-health-risks-of-marble-dust.html (Accessed at 23 April 2021)

[CR26] Gupta SK, Gupta V, Joshi S, Tandon R (2002). Subclinically dry eyes in urban Delhi: an impact of air pollution?. Ophthalmologica.

[CR27] Hegarty DM, Hermes SM, Morgan MM, Aicher SA (2018). Acute hyperalgesia and delayed dry eye after corneal abrasion injury. Pain Reports.

[CR28] Isawumi MA, Adeoti CO, Ubah IN, Oluwatimlelin IO, Raji RA (2011). Ocular status of commercial vehicle drivers in osun state. Nigeria Afr J Medicine Med Sc.

[CR29] Islam SS, Doyle EJ, Velilla A, Martin CJ, Ducatman AM (2000). Epidemiology of compensable work related ocular injuries and illnesses: Incidence and risk factors. J Occup Environ Med.

[CR30] Jeganathan VS, Wang JJ, Wong TY (2008). Ocular associations of diabetes other than diabetic retinopathy. Diabetes Care.

[CR31] Kamel SS, Mohammed TH, El Zankalony YA, Saad AH (2017). Prevalence of dry eye in diabetics. J Egypt Ophthalmol Soc.

[CR32] Kanoff JM, Turalba AV, Andreoli MT, Andreoli CM (2010). Characteristics and outcomes of work-related open globe injuries. Am J Ophthalmol.

[CR33] Koffuor GA, Kyei S, Gyanfosu L (2012). Effect of the working environment on oculo-visual health of some sand and stone miners in Ghana. J Environ Occup Sci.

[CR34] Kuhn F, Morris RE, Witherspoon CD, Mester V (2004). Birmingham Eye Trauma Terminology system (BETT). Journal Français d Ophtalmologie.

[CR35] Mantyjarvl J (2000). The Effect of cataract in traffic. Am J Optom.

[CR36] Mir MD, Jehan A, Qadri SS, Wani RM, Bashir H, Shafi T (2014). An epidemiological study on prevalence and pattern of ocular injuries in kashmir valley–a conflict zone. Int J Med Sci Public Health.

[CR37] Moore EE, Feliciano DV, Mattox KL (2017) Trauma, 8th Edition, Kindle Edition. McGraw-Hill Education. Available at: https://books.google.com.eg/books?id=EwUZnQAACAAJ

[CR38] Ngo CS, Leo SW (2008). Industrial accident-related ocular emergencies in a tertiary hospital in Singapore. Singapore Med J.

[CR39] Occupational safety and Health Administration (OSHA). (2016). Eye and Face Protection-Overview. Available at: http://www.osha.gov/eye-face-protection. Accessed August 2021

[CR40] Occupational safety and health administration (OSHA) (2019). App D - Medical Questionnaires; Mandatory. Available at: https://www.osha.gov/laws regs/regulations/standardnumber/1926/1926.1101AppD (Last updated May 14, 2019).

[CR41] Okoye OI, Umeh RE (2000). Eye health of industrial workers in Southeastern Nigeria. West Afr J Med.

[CR42] Ozdemir, M., Buyukbese, M.A., Cetinkaya, A., and Ozdemir, G. (2003). Risk factors for ocular surface disorders in patients with diabetes mellitus. Diabetes Res Clin Pract 59:195(3)–199. 10.1016/s0168-8227(02)00244-9 10.1016/s0168-8227(02)00244-9 12590016

[CR43] Paz SH, Slotkin J, McKean-Cowdin R (2013). Development of a vision-targeted health-related quality of life item measure. Qual Life Res.

[CR44] Peate W (2007). Work-related eye injuries and illness. Am Fam Physician.

[CR45] Preventblindness (2016). Preventing Eye Injuries. Available from: https://preventblindness.org/preventing-eye-injuries/#1585701834003-65402908-aa1a. (Accessed 15 March 2021)

[CR46] Qayum S, Anjum R, Garg P (2016). Epidemiological pattern of ocular trauma in a tertiary hospital of Northern India. Int J Med Clin Res.

[CR47] Ramesh N, Joseph B (2015). Health and social wellbeing of the workers in the stone quarrying and crushing industry. Int J Med Health Research.

[CR48] Rohatgi S (2013). Pterygium: an epidemiological study in India. Int J Healthcare Biomed Res.

[CR49] Salem HS (2021). Evaluation of the stone and marble industry in Palestine: environmental, geological, health, socioeconomic, cultural, and legal perspectives, in view of sustainable development. Environ Sci Pollut Res.

[CR50] Saxena R, Srivastava S, Trivedi D, Anand E, Joshi S, Gupta SK (2003). Impact of environmental pollution on the eye. Acta Ophthalmol Scand.

[CR51] Sis.gov (2019). Marble industry in Egypt. Economic breakthrough (translated from Arabic) Available from: https://sis.gov.eg/Story/181414/صناعة-الرخام-فى-مصر..انطلاقة-اقتصادية?lang=ar

[CR52] Schaumberg DA, Dana R, Buring JE, Sullivan DA (2009). Prevalence of dry eye disease among US men: estimates from the physicians’ health studies. Arch Ophthalmol.

[CR53] Schiffman RM, Christianson MD, Jacobsen G (2000). Reliability and validity of the Ocular Surface Disease Index. Arch Ophthalmol.

[CR54] Shapiro A, Merin S (1979). Schirmer test and break-up time of tear film in normal subjects. Am J Ophthalmol.

[CR55] Sharma B (2011). Dry eye: demography and attributable risk factors. Post Graduate Medical Journal of NAMS.

[CR56] Shashikala P, Sadiqulla M, Shivakumar D, Prakash KH (2013). Profile of ocular trauma in industries-related hospital. Indian J Occup Environ Med.

[CR57] Shepherd M, Baker R, Scott D, Hockey R, Spinks D, Pitt R (2006). Occupational Eye injuries. Injury Bulletin. Queensland injury surveillance unit. No.90 March. Accessed July 2021

[CR58] Sledge S, Henry C, Borchman D, Yappert MC, Bhola R, Ramasubramanian A, Blackburn R, Austin J, Massey K, Sayied S, Williams A, Georgiev G, Schikler KN (2017). Human Meibum Age, Lipid-Lipid Interactions and Lipid Saturation in Meibum from Infants. Int J Mol Sci.

[CR59] Tetteh, K.K.K., Owusu, R., and Axame, W.K. (2020). "Prevalence and Factors Influencing Eye Injuries among Welders in Accra, Ghana", Advances in Preventive Medicine, vol. 2020, Article ID 2170247, 8. 10.1155/2020/2170247 10.1155/2020/2170247 PMC7516735 33014472

[CR60] Titiyal JS, Falera RC, Kaur M, Sharma V, and Sharma N (2018). Prevalence and risk factors of dry eye disease in North India: Ocular surface disease index-based cross-sectional hospital study. Indian J Ophthalmol; 66:207–11. *Available from:* https://www.researchgate.net/publication/322833514_Prevalence_and_risk_factors_of_dry_eye_disease_in_North_India_Ocular_surface_disease_index-based_cross-sectional_hospital_study. Accessed 20 Sep 2021. 10.4103/ijo.IJO_698_17 PMC5819096 29380759

[CR61] Zhang X, Zhao L, Deng S, Sun X, Wang N (2016). Dry eye syndrome in patients with diabetes mellitus: prevalence, etiology, and clinical characteristics. J Ophthalmol.

